# Dermoscopy of a Lentigo Maligna Less Than 1.5 mm in Diameter

**DOI:** 10.5826/dpc.1102a27

**Published:** 2021-03-08

**Authors:** Karim Saleh

**Affiliations:** 1 Division of Dermatology and Venereology, Department of Clinical Sciences, Lund University, Skåne University Hospital, Lund, Sweden

**Keywords:** lentigo maligna, angulated lines, micromelanoma, dermoscopy

## Introduction

We report the smallest lentigo maligna (LM) detected by physical examination and dermoscopy (1.4 mm in diameter dermoscopically and 1.3 mm histologically) in a 74-year-old woman who presented to our clinic.

## Case Presentation

**Figure 1:**
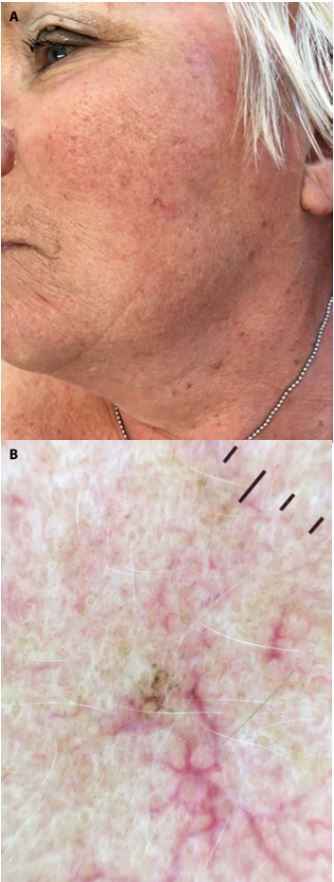
(A) Clinical photograph of the left cheek. (B) Polarized contact dermoscopy of the lesion.

**Figure 2:**
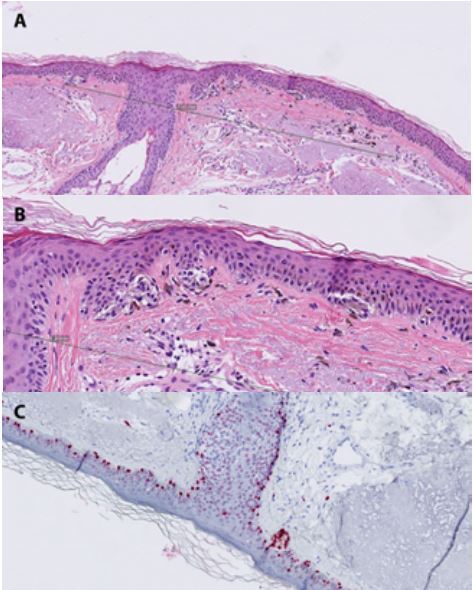
(A) Microscopic sections with H&E stain show an asymmetrical proliferation of melanocytes organized in nests that vary in size and distribution (×10) with a subepithelial actinic elastosis and telangiectatic vessels. Lesion size was 1.3 mm. (B) The same section at ×25. (C) SOX-10 stain illustrating periadnexal extension.

The patient had a history of melanoma on her left arm in 2018. She visited for a regular check-up. She had not been monitored with total body photography. During physical examination a tiny lesion next to telangiectasias on her left cheek was noted. She was not aware of this lesion due to its tiny size. Dermoscopy revealed sun-damaged skin with telangiectasias surrounding a 1.4-mm pattern of light brown dots forming angulated lines (Figure 1). Lentigo maligna was suspected and the lesion excised. Histopathology confirmed the diagnosis of a lentigo maligna (Figure 2).

## Conclusions

Angulated lines in facial lesions are early features that can indicate lentigo maligna [1]. These lines have previously been referred to as the zigzag pattern or rhomboidal structures. Akay et al. [2] documented the smallest melanoma ever published that measured 0.9 mm. However, that lesion was detected using a total-body imaging system. The lesion here is the smallest lesion detected by physical examination without the aid of an imaging system, and to the best of the author’s knowledge, the smallest lentigo maligna lesion ever published.

## References

[1] Schiffner R, Schiffner-Rohe J, Vogt T (2000). Improvement of early recognition of lentigo maligna using dermatoscopy. J Am Acad Dermatol.

[2] Akay BN, Okcu Heper A, Clark S, Erdem C, Rosendahl CO, Kittler H (2017). Dermatoscopy of a melanoma less than one millimeter in diameter. Int J Dermatol.

